# Reduction of Cellular Lipid Content by a Knockdown of *Drosophila PDP1* ****γ**** and Mammalian Hepatic Leukemia Factor

**DOI:** 10.1155/2013/297932

**Published:** 2013-08-22

**Authors:** Svetlana Dzitoyeva, Hari Manev

**Affiliations:** Department of Psychiatry, Psychiatric Institute, University of Illinois at Chicago, Chicago, IL 60612, USA

## Abstract

In exploring the utility of double-stranded RNA (dsRNA) injections for silencing the *PAR-domain protein 1 (Pdp1)* gene in adult *Drosophila*, we noticed a dramatic loss of fat tissue lipids. To verify that our RNAi approach produced the expected *Pdp1* knockdown, the abdominal fat tissues sections were stained with PDP1 antibodies. PDP1 protein immunostaining was absent in flies injected with dsRNA targeting a sequence common to all known *Pdp1* isoforms. Subsequent experiments revealed that lipid staining is reduced in flies injected with dsRNA against Pdp1**γ** (fat body specific) and not against *Pdp1* **ε** (predominantly involved in circadian mechanisms). *Drosophila* PDP1**γ** protein shows a high homology to mammalian thyrotroph embryonic factor (TEF), albumin D site-binding protein (DBP), and hepatic leukemia factor (HLF) transcription factors. In an in vitro model of drug- (olanzapine-) induced adiposity in mouse 3T3-L1 cells, the mRNA content of HLF but not TEF and DBP was increased by the drug treatment. A knockdown of the HLF mRNA by transfecting the cultures with HLF dsRNA significantly reduced their lipid content. Furthermore, the HLF RNAi prevented olanzapine from increasing the cell lipid content. These results suggest that the PDP1/HLF system may play a role in physiological and drug-influenced lipid regulation.

## 1. Introduction

Contrary to the previous belief that in adult *Drosophila* (fruit fly) RNA interference (RNAi) cannot occur by exogenous administration of double-stranded RNA (dsRNA), it was conclusively demonstrated that this type of systemic RNAi is operative in adult flies [1]. This mechanism explains the efficacy of dsRNA injections into adult *Drosophila*, applied as a tool for targeted gene knockdown in adult organisms [2–4]. The advantage of this RNAi method is that it avoids the unwanted developmental alterations and the possible side effects of genetic manipulations involved in alternative RNAi approaches. In the course of exploring the utility of dsRNA injections for silencing the *PAR-domain protein 1* (*Pdp1*) gene in adult *Drosophila*, we noticed a peculiar RNAi-related phenotype, a dramatic loss of fat tissue lipids. Here, we report the follow-up study aimed at exploring this serendipitous discovery.

*Drosophila Pdp1 *encodes a transcription factor highly homologous to the proline- and acidic amino acid-rich (PAR) subfamily of mammalian bZIP transcription factors, albumin D site-binding protein (DBP), hepatic leukemia factor (HLF), and thyrotroph embryonic factor (TEF). PDP1 was originally identified as a regulator of the muscle activator region [5]. Subsequently, it was established that *Pdp1* is a component of the *Drosophila* circadian network and that its expression is directly activated by *dClock/Cycle* genes [6, 7]. The *Pdp1* gene encodes multiple transcripts, which are differentially expressed during embryogenesis [8]. Of the six *Pdp1* isoforms, *Pdp1*ε**is the one that shows a circadian expression and is involved in the regulation of the *Drosophila* circadian behavior [9]. Whereas *Pdp1*ε** is predominantly expressed in the nervous system, *Pdp1*γ** is predominantly expressed in the fat body [8]. The mammalian homologous factors DBP, TEF, and HLF also show a circadian rhythm in their expression/accumulation; their absence results in epilepsy [10]. Furthermore, these mammalian proteins have recently been linked to a mechanism of fatty acid regulation [11]. To address possible similarities between *Drosophila Pdp1* and its mammalian homologues, in addition to the experiments in fruit flies, we employed a model of drug-induced adiposity in mammalian 3T3-L1 cells in vitro [12]. 

## 2. Material and Methods

### 2.1. Drosophila and Cell Culture

Male Canton-S flies were cultured for 5–7 days in a 12 h/12 h light/dark cycle [3]. They were CO_2_ anesthetized, injected with 0.3-0.4 pmol dsRNA [3], and collected five days later. Mouse 3T3-L1 cells (American Type Culture Collection), grown to confluence in Dulbecco's modified Eagle's medium (GIBCO)/10% fetal bovine serum (Atlanta Biologicals), were differentiated into adipocytes by changing to a differentiation medium [12]. dsRNA transfection (10 pmol; TransPass R1 transfection reagent; New England Biolabs) started 24 h after addition of the differentiation medium; cells were collected 48 h later. Olanzapine (50 *μ*M; Sequoia Research Products Ltd.) and vehicle (1 : 1000 dimethyl sulfoxide; Sigma) were added either together with the differentiation medium or 24 h after the initiation of dsRNA transfection.

### 2.2. Double-Stranded RNA (dsRNA)

For *Drosophila* dsRNA RNAi, the corresponding RNAs were synthesized in vitro from DNA oligonucleotides with an attached T7 RNA polymerase promoter sequence (IDT DNA Technologies) targeting the aacttccctcctcagttgccgg cDNA region common to all *Pdp1* isoforms (AF172402, AF172403, AF172404, AF172405, AF172406, and AF209903), the agttacgcgttgttgctgcgacc cDNA region unique to *Pdp1*γ** (AF172404), and the aacgtaccagaggatttaccagg region unique to *Pdp1*ε** (AF172406). Control dsRNA was based on the human 5-lipoxygenase cDNA, ttcatgcacatgttccagtctt (NM_000698; no matches to *Drosophila* genome sequences; *Drosophila* does not have 5-lipoxygenase homologues). The single-stranded sense and antisense DNA oligonucleotides were annealed in distilled water and 100 ng was used in 30 *μ*L of in vitro transcription reaction. dsRNA molecules were formed by combining the corresponding transcribed sense and antisense RNAs (10 min at 65°C) [2, 3]. For mouse 3T3-L1 dsRNA RNAi, a 154 nucleotide fragment (1120–1274) of mouse HLF mRNA (NM_172563) was cloned into a pGEM-T vector (Promega). The insert was amplified with M13 primers and RNA was transcribed in vitro with T7 and SP6 RNA polymerases (New England Biolabs). An equal amount of the single-stranded sense and antisense RNA molecules was incubated for 10 min at 65°C and cooled on ice. Control dsRNA was prepared from a 239-nucleotide fragment (394–620) of the green fluorescent protein gene (X83959) amplified from the pGFP cloning plasmid DNA vector.

### 2.3. mRNA Assay

RNA was extracted from cells and *Drosophila* homogenates with TRIzol reagent (Invitrogen), reverse transcribed, and used for the quantitative real time PCR (Stratagene Mx3005P qPCR System; Agilent Technologies with Maxima SYBR Green qPCR Master Mix (MM); Fermentas Inc.), in a two-step PCR program [14]: the reaction mix: 10 *μ*L 2x MM, 2 *μ*L primer mix (0.2 *μ*M final, each), and 8 *μ*L reverse transcription mix. The corresponding primers are shown in Table 1. Data were normalized against the cyclophilin (3T3-L1 cells) or RP49 (*Drosophila*) internal control and presented as a coefficient of variation [14].

### 2.4. PDP1 Immunostaining

30-micron *Drosophila* cryosections were fixed in 12% formaldehyde/phosphate buffered saline (PBS), rinsed in PBS, and incubated with PDP1 antibody (1 : 500, overnight, 4°C) [8] followed by incubation with secondary biotinylated anti-rabbit IgG (1 h; Vector Laboratories). Color was developed with DAB (Sigma).

### 2.5. Lipid Staining and Quantification

For lipid visualization in the *Drosophila* fat body, whole fly 30-micron cryosections were fixed, incubated with Oil Red O (ORO; Sigma; 0.5% in isopropanol diluted with water 3 : 2 and filtered through a 0.45 *μ*m filter) for 30 min, rinsed, covered with 80% glycerol, and photographed. For quantitative measurements, flies were decapitated and their bodies cryosectioned and fixed. Free-floating sections (from 5 bodies/data point) were stained with ORO, washed, and dried, and the dye was recovered with isopropanol. 3T3-L1 cells were fixed and incubated (30 min) with ORO. After discarding this solution, the dye captured by intracellular lipids was recovered with 400 *μ*L isopropanol, and the absorbance was measured (single wavelength, 520 nm filter; Bio-Rad, Model 550). The results are reported in units.

### 2.6. Statistics

Statistical analyses were performed using SPSS software (SPSS Inc.). Data (mean ± SEM) were analyzed by one-way analysis of variance followed by Student's *t*-test or Dunnett's test (significance at *P* < 0.05).

## 3. Results

### 3.1. Drosophila Pdp1 dsRNA RNAi

**Figure 1:**
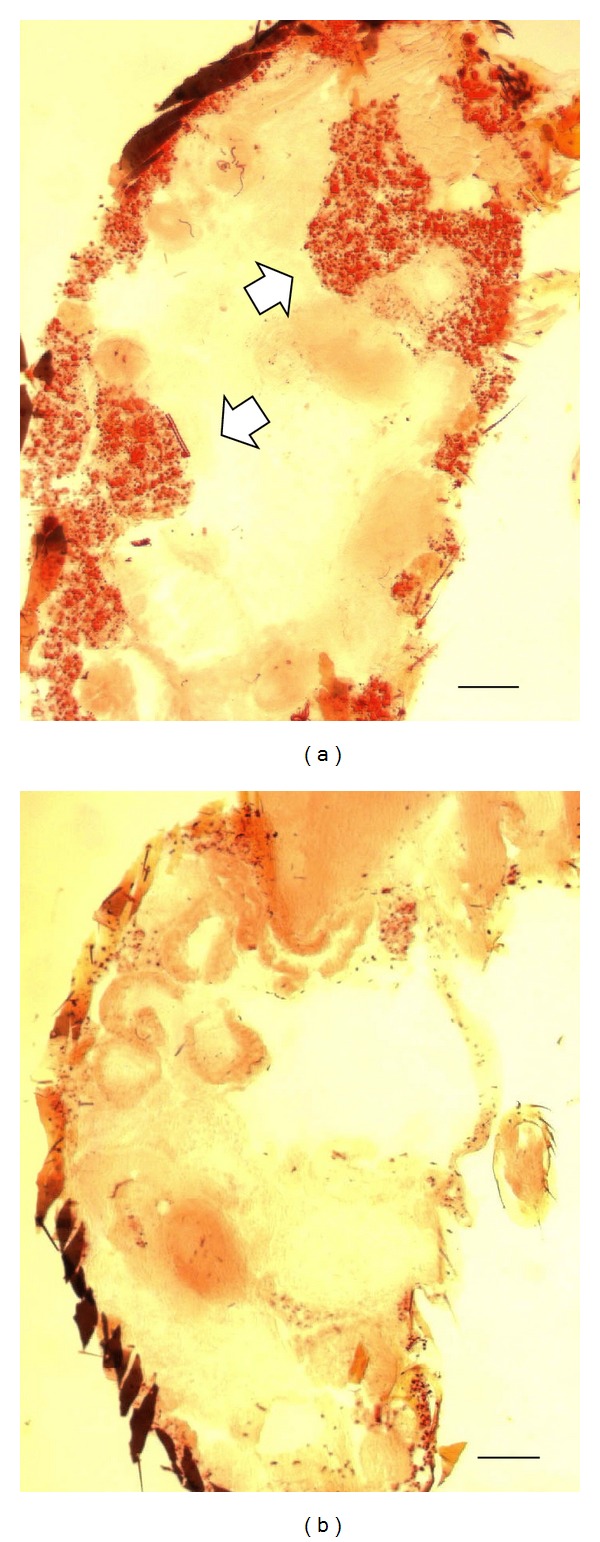
Effect of total *Pdp1* RNAi induced in adult *Drosophila* on lipid staining. Flies were injected with control dsRNA (panel (a)) and dsRNA targeted at a sequence common to all known *Pdp1* isoforms (total *Pdp1* RNAi; panel (b)). They were processed for lipid staining (red) five days later. Shown is a low magnification (objective 5x) sagittal abdominal section (size bar = 150 *μ*m). Note the presence of lipid staining in the fat tissue cells of a control fly (indicated by white arrows) (a) and its absence in the *Pdp1* RNAi fly (b).

**Figure 2:**
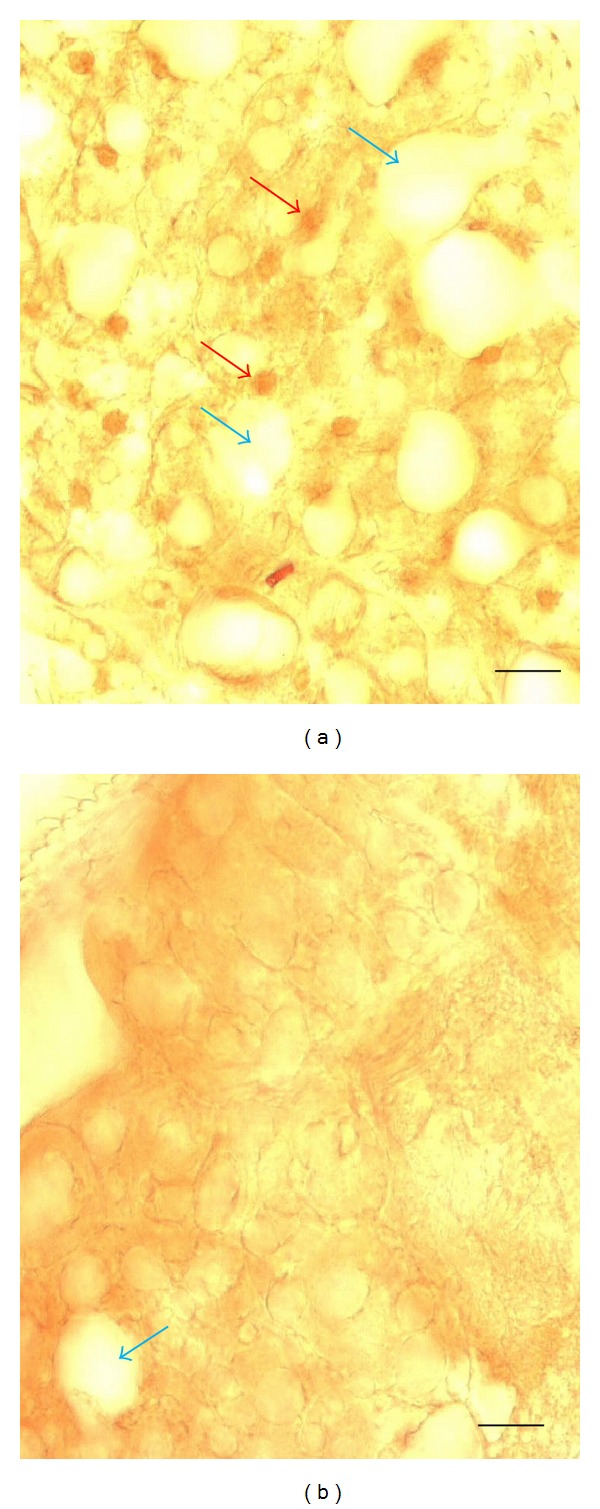
Effect of total *Pdp1* RNAi induced in adult *Drosophila* on PDP1 protein immunostaining in the fat tissue. Flies were injected with control dsRNA (panel (a)) and dsRNA targeted at a sequence common to all known *Pdp1* isoforms (total *Pdp1* RNAi; panel (b)). They were processed for PDP1 immunolabeling five days later. Shown is a high magnification (objective 20X) of abdominal fat tissue section (size bar = 20 *μ*m). Note the presence of strong nuclear PDP1 protein immunolabeling (dark circles indicated by red arrows) in the control section (a) and its absence in the *Pdp1* RNAi section (b). Also, indicated (blue arrows) are multiple white circles of lipid droplet-containing cells in the control section and their reduced number after *Pdp1* RNAi.

Our initial observation of the lipid-reducing effect of *Pdp1* RNAi was made by examining fly body sections without the help of any lipid staining; that is, the effect was rather obvious. To verify this observation, we employed the ORO lipid staining. In these experiments, a total *Pdp1* RNAi was achieved by injecting flies with the dsRNA targeted at a sequence common to all known *Pdp1* isoforms. This resulted in the loss of ORO lipid staining as exemplified by the staining of *Drosophila* abdominal sections (Figure 1). To verify that our RNAi approach produced the expected *Pdp1* knockdown, we stained the whole fly sections including the abdominal fat tissue with PDP1 protein antibodies. PDP1 immunostaining revealed a robust nuclear PDP1 staining in the cells of flies injected with control dsRNA and the absence of PDP1 staining in *Pdp1* dsRNA injected flies (Figure 2). Also, in these sections, we confirmed the lipid-reducing effect of the total *Pdp1* dsRNA; the number and the size of lipid-containing droplets in fat tissue cells were reduced in the RNAi samples (Figure 2).

**Figure 3:**
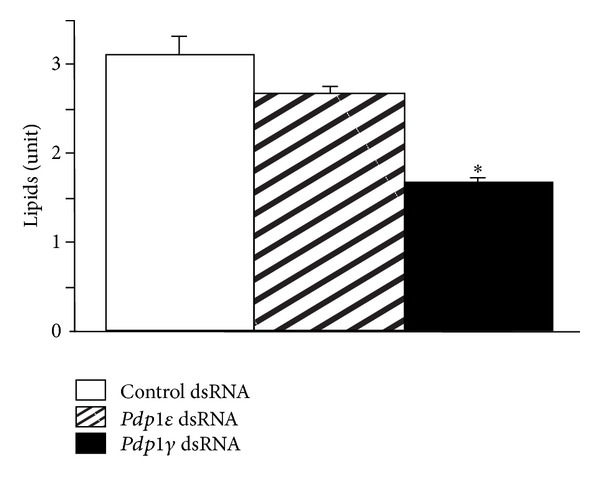
Quantitative assay of *Drosophila* lipid content following the isoform-specific *Pdp1* RNAi. Adult flies were injected with control, *Pdp1*γ**, and *Pdp1*ε** dsRNA and processed for quantitative lipid assay. *Pdp1*γ** but not *Pdp1*ε** dsRNA reduced body lipid content (*P < 0.001 versus control; *n* = 5; mean ± standard error mean).

The previous results were obtained with the dsRNA targeted at a sequence common to all known *Pdp1* isoforms. In subsequent experiments, we investigated the *Pdp1* isoform specificity of the lipid-reducing phenotype. Hence, flies were injected with dsRNA targeted specifically against *Pdp1*ε** (predominantly present in the nervous system and involved in circadian mechanisms) and *Pdp1*γ** (expressed in the fat body), respectively, and their body lipid content was quantified. Only the *Pdp1*γ** dsRNA significantly reduced the body lipid content (Figure 3).

### 3.2. Mammalian HLF dsRNA RNAi

**Figure 4:**
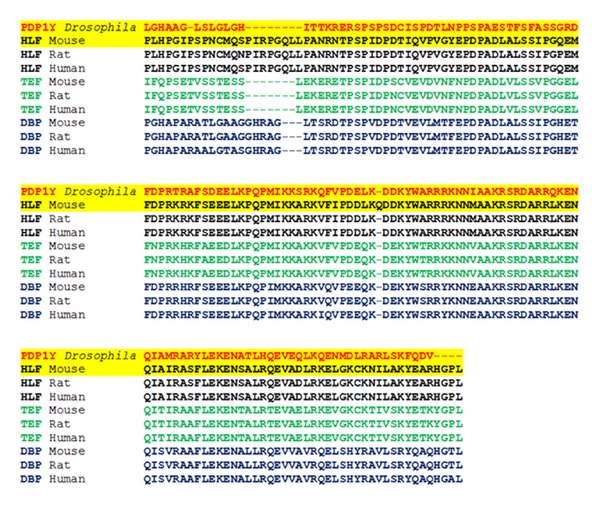
Homology between *Drosophila *PDP1*γ* and mammalian bZIP transcription factors HLF, TEF, and DBP. Shown is the protein sequence alignment in the b-ZIP structural regions with the highest homology, analyzed using the online CLUSTALW multiple sequences alignment tool [13]. The NCBI database accession information is as follows. *Drosophila* PDP1*γ* Q9TVQ4; HLF: mouse NP_766151, rat Q64709, and human NP_002117; TEF: mouse NP_059072, rat NP_062067, and human NP_003207; DBP: mouse NP_058670, rat NP_036675, and human NP_001343. Highlighted in yellow are the sequences of *Drosophila* PDP1*γ* and mouse HLF.

**Figure 5:**
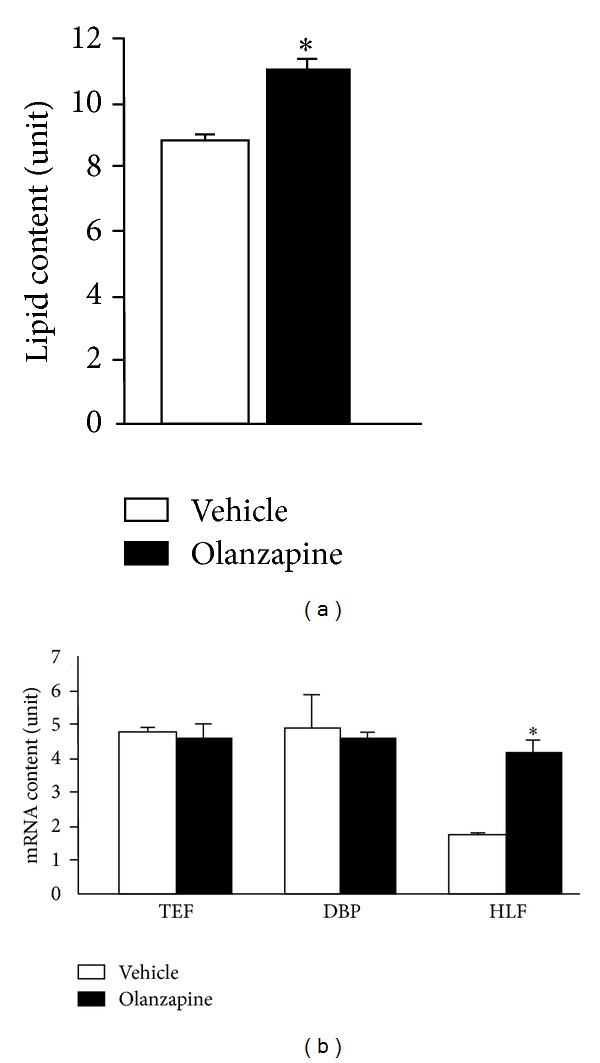
Effect of olanzapine on lipid content (a) and mRNA content of mammalian *Pdp1 *homologues (b) in mouse 3T3-L1 cells. Vehicle (open bars) and olanzapine (50 *μ*M; closed bars) were present in the culture medium for 24 h. Olanzapine increased lipid content (a) and HLF mRNA content (normalized to internal control mRNA) (b) (*P < 0.001 versus corresponding control; *n* = 6; mean ± standard error mean).

**Figure 6:**
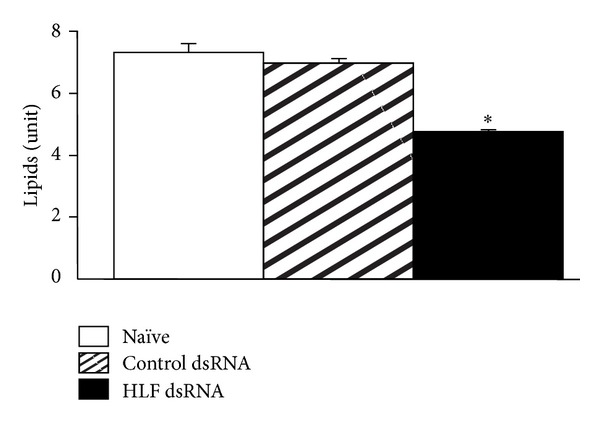
Effect of mammalian HLF RNAi on lipid content in mouse 3T3-L1 cells. Cells in culture were transfected with control and HLF dsRNA and processed for lipid assay 48 h later (see text for details). Lipid content in 3T3-L1 cells was reduced by HLF dsRNA treatment (*P < 0.001 versus naïve; *n* = 6; mean ± standard error mean), which also reduced HLF mRNA content (not shown).

**Figure 7:**
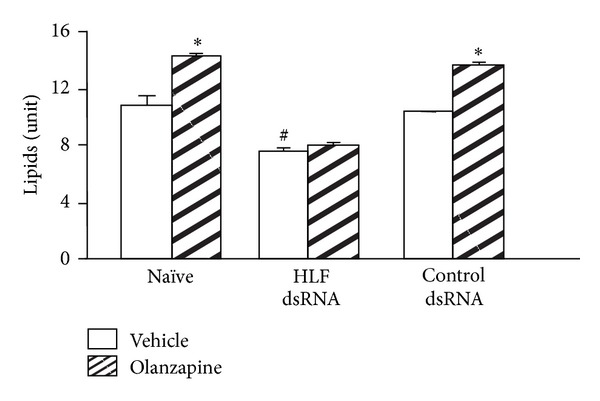
HLF RNAi prevents the adipogenic effect of olanzapine in mouse 3T3-L1 cells. Olanzapine and vehicle treatments were initiated 24 h after the initiation of transfection and conducted for the next 24 h. At the end of the treatment, cells were assayed for lipid content (*P < 0.001 versus corresponding vehicle; ^#^ *P* < 0.001 versus naïve and control dsRNA transfection; *n* = 3; mean ± standard error mean).

*Drosophila *PDP1*γ* protein shows a high homology to TEF, DBP, and HLF members of the PAR subfamily of mammalian bZIP transcription factors (Figure 4) [13]. To explore possible similarities between *Pdp1*γ** and these factors, we selected the mouse 3T3-L1 preadipocytes in vitro. These cells have been used as a model for drug-induced adipogenic effects; that is, treatment of 3T3-L1 cells during differentiation into adipocytes with the antipsychotic drug olanzapine increases their lipid content [12]. In this model, we found that the adipogenic olanzapine treatment increases the mRNA content of HLF but not TEF and DBP (Figure 5). On the other hand, a knockdown of the endogenous HLF mRNA by transfecting the cultures with HLF dsRNA significantly reduced their lipid content (Figure 6). In an experiment in which olanzapine and vehicle treatments were initiated 24 h after the initiation of transfection and conducted for the next 24 h, we found that olanzapine treatment increased lipid content in naïve and sham-dsRNA transfected cells but not in HLF dsRNA-transfected cells (Figure 7).

## 4. Discussion

In this work, we confirmed and expanded our serendipitous observation that a systemic PDP1 knockdown in adult flies, induced by injections of *Pdp1* dsRNAs, leads to a significant lipid decrease, and we found that similar phenotype can be induced by HLF RNAi in mouse adipocytes in vitro. Collectively, our results demonstrated that a reduction of the PDP1*γ*/HLF transcription factor leads to a decreased lipid content.

Both mammalian HLF and *Drosophila* PDP1 are known components, that is, output regulators, of circadian cycles and as such have been linked to metabolic regulation [15]. The expression of *Drosophila* circadian genes (i.e., peripheral clocks) in the fat body has been shown to play a role in the regulation of fly metabolism [16]. Of the six *Pdp1* isoforms, *Pdp1*ε** is the one that is characterized by a prominent circadian expression and is involved in the regulation of the *Drosophila* circadian behavior [9]. One of the circadian functions of *Pdp1*ε** is in regulating the circadian output gene *takeout* [17]. In male *Drosophila*, *takeout* is abundant in the fat body and plays a role in courtship behavior of these flies [18]. It was suggested that *Pdp1*ε**-mediated regulation of the fat body genes may influence this type of fly behavior [17]. In our *Drosophila* experiments, lipid staining was decreased by *Pdp1*γ** and not *Pdp1*ε** RNAi. Hence, it would be interesting to elucidate whether in addition to *Pdp1*ε** also* Pdp1*γ** regulates the expression of the output genes such as *takeout* and whether this mechanism mediates the observed lipid-decreasing effects of* Pdp1*γ** knockdown.

Our experiments with adipocytes show that in addition to systemic PDP1/HLF alterations (e.g., systemic PDP1 knockdown in adult flies), also direct cellular HLF alterations (e.g., knockdown in 3T3-L1 cells) can reduce lipid content.

In our in vitro experiments, a drug-induced adiposity was accompanied by increased levels of HLF mRNA, whereas HLF RNAi was accompanied by decreased lipid content. Furthermore, it was previously reported that in a mouse model of severe reduction of lipid accumulation and severe loss of body weight, the liver HLF mRNA levels, along with the TEF and DBP mRNA levels, are significantly reduced [19]. Hence, HLF appears to be involved in the physiological regulation of cellular lipid levels.

In our experimental conditions, the RNAi-mediated HLF mRNA reduction significantly diminished a drug -induced adiposity (i.e., olanzapine). Olanzapine belongs to a class of drugs known as the second generation antipsychotic drugs (SGADs). All these compounds are capable of triggering significant weight gain associated with adverse metabolic alterations [20, 21]. It has been proposed that these side effects may occur by a direct stimulatory action of SGADs on adipocytes [12, 22, 23]. Our in vitro experiments confirmed the direct adipogenic action of olanzapine and found that this action can be diminished by HLF reduction. In the therapy of psychiatric patients with SGADs, a better understanding of the mechanisms that lead to metabolic side effects is needed to identify the risk factors that facilitate and exacerbate this SGADs-associated clinical problem. Our results suggest for the first time that the HLF pathway could be such a mechanism. Furthermore, the observed direct susceptibility of the adipocyte HLF and lipids to regulation by drugs (e.g., olanzapine-increased HLF mRNA and lipid contents) suggests that future pharmacological tools could be tailored specifically to the adipocyte HLF pathway to interfere therapeutically with the mechanisms of adiposity.

## Figures and Tables

**Table 1:** Primers used for the mRNA assay.

Target	Forward	Reverse
HLF	5′-gaaggagctgggcaaatgcaagaa-3′	5′-accagacaggaaacaagctgtcca-3′
Cyclophilin	5′-agcatacaggtcctggcatcttgt-3′	5′-aaacgctccatggcttccacaatg-3′
Pdp1 total	5′-acttccctcctcagttgccgg-3′	5′-tcgcagatggtctgtgtgta-3′
Pdp1*ε*	5′-ccacgctaacgtaccagaggattt-3′	5′-ctgaaatcgcgctttcaagctgtt-3′
Pdp1*γ*	5′-gagtttcagttacgcgttgttgct-3′	5′-gttgttctccgacaggaactcgtc-5′
RP49	5′-atgaccatccgcccagcataca-3′	5′-tgtgtattccgaccaggttac-3′

## Abbreviations

DBP: Albumin D site-binding protein
dsRNA: Double-stranded RNA
HLF: Hepatic leukemia factor
PAR: Proline- and acidic amino acid rich
PDP1: PAR-domain protein 1
RNAi: RNA interference
SGADs: Second generation antipsychotic drugs
TEF: Thyrotroph embryonic factor.
